# Protective effects of butyrate on cerebral ischaemic injury in animal models: a systematic review and meta-analysis

**DOI:** 10.3389/fnins.2024.1304906

**Published:** 2024-02-29

**Authors:** Shichang Yan, Qipei Ji, Jilin Ding, Zhixiang Liu, Wei Wei, Huaqiang Li, Luojie Li, Chuan Ma, Defu Liao, Ziyan He, Shuangchun Ai

**Affiliations:** ^1^School of Health and Rehabilitation, Chengdu University of Traditional Chinese Medicine, Chengdu, China; ^2^Department of Rehabilitation, Mianyang Hospital of Traditional Chinese Medicine, Mianyang, China; ^3^School of Pharmacy, Chengdu University of Traditional Chinese Medicine, Chengdu, China

**Keywords:** ischaemic stroke, butyrates, inflammation, apoptosis, meta-analysis, microbial metabolites

## Abstract

**Introduction:**

Cerebral ischaemic stroke is a common disease that poses a serious threat to human health. Butyrate is an important metabolite of intestinal microorganisms. Recent studies have shown that butyrate has a significant protective effect in animal models of cerebral ischaemic injury.

**Objective:**

The aim of this study was to evaluate the protective effect of butyrate on cerebral ischaemic stroke by meta-analysis, aiming to provide a scientific basis for the clinical application of butyrate in patients with cerebral ischaemia.

**Materials and methods:**

A systematic search was conducted for all relevant studies published before 23 January 2024, in PubMed, Web of Science, Cochrane Library, and Embase. Methodological quality was assessed using Syrcle’s risk of bias tool for animal studies. Data were analysed using Rev Man 5.3 software.

**Results:**

A total of nine studies were included, and compared with controls, butyrate significantly increased BDNF levels in the brain (SMD = 2.33, 95%CI = [1.20, 3.47], *p* < 0.005) and P-Akt expression (SMD = 3.53, 95% CI = [0.97, 6.10], *p* < 0.05). Butyrate also decreased IL-β levels in the brain (SMD = −2.02, 95% CI = [−3.22, −0.81], *p* < 0.005), TNF-α levels (SMD = −0.86, 95% CI = [−1.60, −0.12], *p* < 0.05), and peripheral vascular IL-1β levels (SMD = −2.10, 95%CI = [−3.59, −0.61], *p* < 0.05). In addition, butyrate reduced cerebral infarct volume (MD = −11.29, 95%CI = [−17.03, −5.54], *p* < 0.05), mNSS score (MD = −2.86, 95%CI = [−4.12, −1.60], *p* < 0.005), foot fault score (MD = −7.59, 95%CI = [−9.83, −5, 35], *p* < 0.005), and Morris water maze time (SMD = −2.49, 95%CI = [−4.42, −0.55], *p* < 0.05).

**Conclusion:**

The results of this study indicate that butyrate has a protective effect on cerebral ischaemic stroke in animal models, and the mechanism is related to reducing inflammation and inhibiting apoptosis. It provides an evidence-based basis for the future clinical development of butyrate in the treatment of ischaemic stroke.

**Systematic Review Registration:**

https://www.crd.york.ac.uk/PROSPERO/, CRD42023482844.

## 1 Introduction

Stroke is a disease with high incidence and disability. Of these, ischaemic stroke (IS), which involves central nervous system infarction and a systemic inflammatory response, accounts for 71% of all strokes globally (Sacco et al., 2013; Lindsay et al., 2019). The disability-adjusted life-years (DALYs) attributable to IS are 51.9 million per year (Campbell et al., 2019), underscoring the enormous economic and social impact of the disease. A tissue plasminogen activator (tPA) is commonly used in the clinical treatment of IS to break down the clot, thereby chemically achieving rapid reperfusion (Fukuta et al., 2017). However, tPA therapy is effective in less than one-third of patients due to the strict time window requirements (Saver and Levine, 2010). Moreover, tPA only restores cerebrovascular blood flow and does not target neuronal cell damage after IS. Therefore, exploring a safe and effective treatment for IS is urgently needed.

The complex interactions between the gut microbiota and the brain have received widespread attention recently. It has been established that dysregulation of the central nervous system affects the gastrointestinal tract through the nervous, endocrine, and immune systems (O'Mahony et al., 2015). IS can lead to abnormalities in the composition and function of the gut microbiota (Li et al., 2019). For example, recent studies have found that IS affects gut microbial composition and abundance in mice by stimulating the release of norepinephrine from the autonomic nervous system and altering the number of intestinal mucin-producing cuprocytes in mouse models (Huang et al., 2021). In addition, in a porcine model, a significant decrease in the diversity of gut microbes occurred after IS, with the extent of the reduction correlating with the severity of IS (Jeon et al., 2020). Among the changes in phyla, the gut microbiota mainly showed an increase in the phylum Anabaena and a decrease in the phylum Ascomycota (Benakis et al., 2016). At the same time, disordered gut microbes and their metabolites have an essential impact on the pathogenesis of IS (Xia et al., 2019). The study suggests gut microbes can ameliorate post-IS inflammation via T cells (Malkki, 2016; Ridler, 2016). Short-chain fatty acids (SCFAs), a metabolite of gut microbes, also play an essential role in IS. One study found that acetic acid concentrations were significantly lower and valeric acid concentrations were significantly higher in IS patients than in controls. However, total organic acid concentrations were lower in stroke patients (Yamashiro et al., 2017). Butyrate is a particularly crucial signalling molecule among the SCFAs. Chen et al. found that SCFAs were low in IS mice, especially butyrate (Chen et al., 2019). In humans, butyrate is produced from dietary fibre by bacterial fermentation. It is rapidly absorbed by colonic cells by passive non-ionic diffusion or active carrier transport (Nedjadi et al., 2014). It is metabolised mainly in intestinal epithelial cells, hepatocytes, and other tissue cells (Wong et al., 2006). It is of widespread interest because of its regulation of maintaining epithelial integrity. Many animal experiments have reported the neuroprotective effects of butyrate on IS. However, no relevant clinical investigations have been reported. Therefore, this study aimed to systematically analyse these animal experiments to evaluate therapeutic effects of butyrate and provide adequate evidence-based medical support for the clinical application of butyrate in treating IS.

## 2 Materials and methods

A systematic review and meta-analysis of animal studies was performed according to PRISMA guidelines (Tam et al., 2019). The programme is registered in the International Prospective Systematic Evaluation Register (PROSPERO) (registration number CRD42023482844).

### 2.1 Search strategy

Two investigators independently searched PubMed, Web of Science, Cochrane Library, and Embase to collect experimental data on the therapeutic effects of butyrate in an animal model of cerebral ischaemic injury published on 23 January 2024 and disputed by a third investigator. The subject terms “butyrate” or “butyric acid” and “brain infarction,” “brain ischemia,” “cerebral thrombosis,” “cerebral embolism,” or “middle cerebral artery.” or “middle cerebral artery occlusion,” or “Stoke.”

### 2.2 Inclusion and exclusion criteria

The inclusion criteria were pre-specified as follows: (1) the type of study was an animal RCT experiment; (2) animals were used as the study subjects, and a cerebral ischaemia model was established; (3) the experimental group was given butyrate intervention; (4) the main regression indexes were inflammatory factors, BDNF, P-Akt, cerebral infarct volume, and the Neurological Scale Score.

The exclusion criteria were predetermined as follows: (1) non-RCT experiments; (2) non-animal models of cerebral ischaemia; (3) non-butyrate treatments or combinations of butyrate and other treatments; (4) reviews, conferences, and clinical trials; (5) studies with incomplete data, including unpublished data; (6) repetitively published studies; and (7) non-English literature.

### 2.3 Data extraction

Two researchers independently screened the literature using Endnote software. In case of disagreement, a third researcher resolved the dispute. The main contents of data extraction were as follows: (1) General information, including the title of the study, the name of the first author, the year of publication, the type of animal, weight, and model. (2) Interventions, including drug name, dose, mode, and administration time. (3) Outcome-related indicators and data. (4) For the literature that reported data only in images, we extracted the data from the images by using GetData Graph Digitizer software.

### 2.4 Quality assessment

The risk of bias tool from SYRCLE was used to judge each entry as low risk, high risk, and unclear based on appropriate criteria and to create a risk of bias map (Hooijmans et al., 2014).

### 2.5 Data analysis

Data were analysed using Rev Man 5.3 software. Continuous variables were used to calculate their 95% confidence intervals (95% CI) using weighted mean difference (WMD) or standardised mean difference (SMD). Depending on the heterogeneity test, it was treated with a fixed-effects model or a random-effects model. A fixed-effects model was used when the heterogeneity test results of the included studies were *p* > 0.05 and *I*^2^ < 50%; a random-effects model was used when *p* < 0.05 and *I*^2^ ≥ 50%. Sensitivity analysis was used to explore the causes of heterogeneity. Funnel plots were used to assess potential publication bias.

## 3 Results

### 3.1 Research data

**Figure 1:**
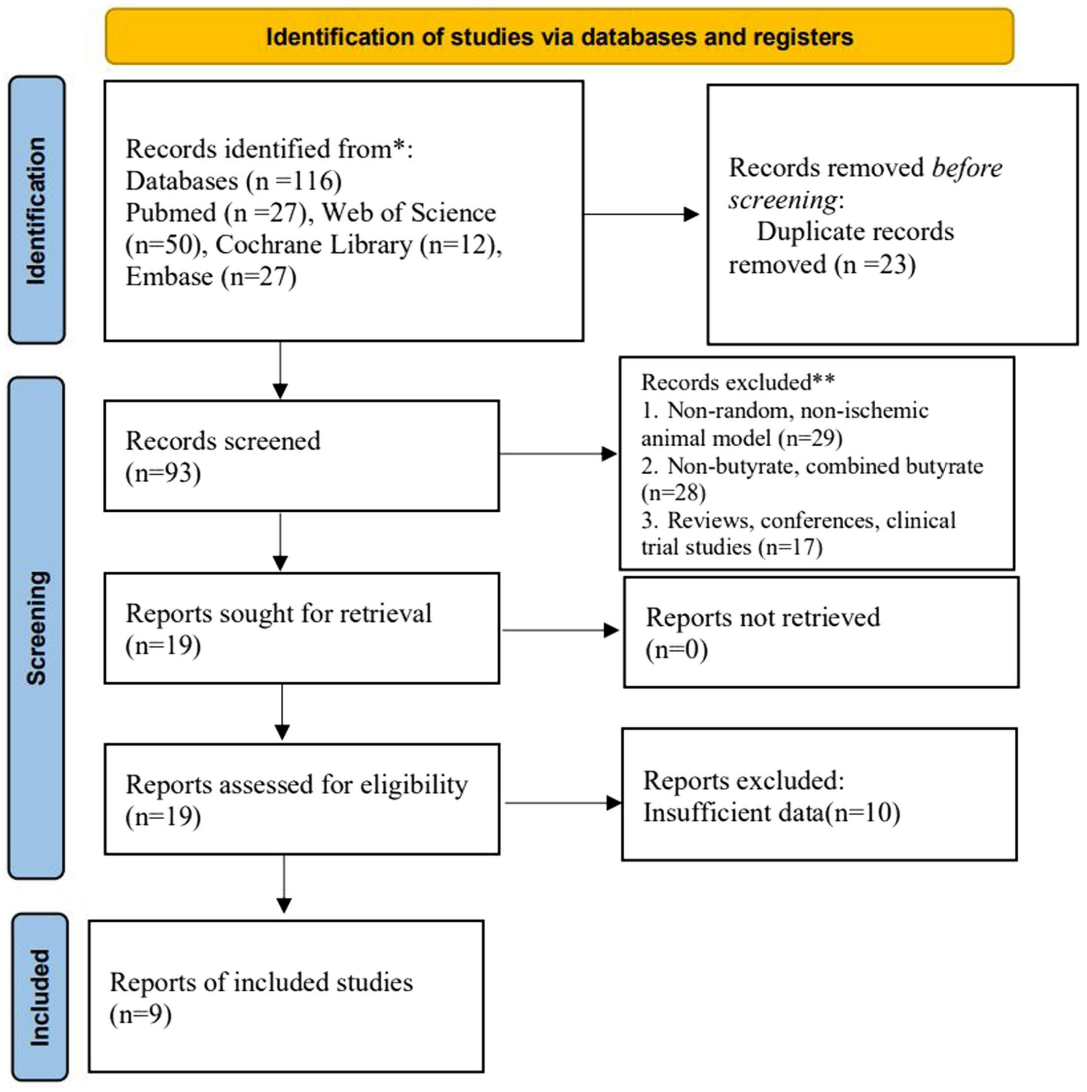
Project for Reporting of Systematic Evaluations and Meta-Analyses (PRISMA) flowchart.

A total of 116 relevant studies were searched. According to the inclusion criteria, 23 duplicates were excluded, and 74 studies that did not meet the requirements of experimental subjects, interventions, and experimental methods were deleted by reviewing study titles and abstracts. In total, 19 articles were reassessed through full-text reading. Of these, valid data were not available for 10 articles. Finally, nine studies were included for meta-analysis (Figure 1).

### 3.2 Basic research information

A total of nine studies were included in this study. The five (Kim et al., 2007; Park and Sohrabji, 2016; Tu et al., 2017; Chen et al., 2019; Zhou et al., 2021) studies were SD rats with a body weight range of 220–360 g. In total, four (Kim et al., 2007; Tu et al., 2017; Chen et al., 2019; Zhou et al., 2021) studies used animals that were males and one (Park and Sohrabji, 2016) study used animal that was a female; the animals used in two (Chen et al., 2023; Li et al., 2023) studies were male C57BL/6 mice with a range of body weights that were not reported; the one (Sun et al., 2015) study used animals that were male ICR mice with a weight range of 22–24 g; the animals used in 1 (Wang et al., 2021) study were male Dd mice, and the body weights of the mice were not reported; nine animal models of occluded middle cerebral arteries were used, including seven MCAO models, one photothrombotic stroke model, and one cerebral I/R injury model. Of the nine studies regarding the control modality, saline control intervention was used in 6 studies, Nacl control intervention was used in 1 study, and 2 studies were not reported. Regarding the mode of butyrate administration, drinking water was used in 2 studies, force-feeding was used in 2 studies, intraperitoneal injection was used in 3 studies, nasal delivery was used in 1 study, and 1 study was unspecified. The specific essential characteristics of the included studies are shown in Table 1.

**Table 1:** Basic characteristics of the included studies.

Included study	Animal characteristics	Animal model	Group (Drugs, Dosage, Route)	Intervention time	Outcome indicators	Results
Chen et al., 2019	Male Sprague Dawley rats (240 ± 20 g, 9 weeks old)	MCAO	C: NS, NR, gavage E: Butyric acid, 30 mg/kg, gavage	24 h after reperfusion and once a day for 14 d	1. Cerebral infarction volume	1. ↓, *p* < 0.05
Li et al., 2023	Wild-type male C57BL/6 mice (8–10 weeks old)	MCAO	C: NR, NR, NR E: SB, NR, NR	After the surgery, once a day for 28 d	1. Cerebral infarction volume 2. mNSS 3. Foot fault 4. Morris water maze 5, 6. IL-1β, TNF-α in brain 7. IL-1β in blood 8. BNDF	1. ↓, *p* < 0.05 2. ↓, *p* < 0.05 3. ↓, *p* < 0.05 4. ↓, *p* < 0.05 5. ↓, *p* < 0.05 6. ↓, *p* < 0.05 7. ↓, *p* < 0.05 8. ↑, *p* < 0.05
Park and Sohrabji, 2016	Female Sprague Dawley rats (9–11 months, 280–360 g)	MCAO	C: NS, NR, NR E:SB, 300 mg/kg, i.p.	6 h after reperfusion and 30 h after cerebral ischemia	1. IL-1β in brain 2. IL-1βin blood	1. ↓, *p* < 0.05 2. ↓, *p* < 0.01
Sun et al., 2015	Male ICR mice (6-week -old, 22–24 g)	Cerebral I/R injury model	C: NS, 5/10 mg/kg, gavage E: SB, 5/10 mg/kg, gavage	3 h after the surgery	1. IL-1β in brain 2. TNF-α in brain 3. BDNF 4. P-AKt	1. ↓, *p* < 0.01 2. ↓, *p* < 0.05 3. ↑, *p* < 0.01 4. ↑, *p* < 0.05
Wang et al., 2021	6 week-old male db/db (C57BLKS/J-m1/1Leprdb/db; Db) mice	MCAO	C: Nacl, 0.1 mol/L, drink E: SB, 0.1 mol/L, drink	After the surgery	1. Cerebral infarction volume 2. IL-1βin blood	1. ↓, *p* < 0.05 2. ↓, *p* < 0.01
Zhou et al., 2021	Adult male Sprague Dawley rats (250–300 g)	MCAO	C: NS, 2.5/7.5/22.5 mg/kg, nasal delivery E: SB, 2.5/7.5/22.5 mg/kg, nasal delivery	1 h after the surgery	1. Cerebral infarction volume 2. Foot fault 3. Morris water maze 4. P-A Kt	1. ↓, *p* < 0.01 2. ↓, *p* < 0.05 3. ↓, *p* < 0.01 4. ↑, *p* < 0.05
Chen et al., 2023	Aged male C57BL/6 mice (17–19 months)	Photothrombotic stroke model	C: NR, NR, NR E: Butyrate, NR, drink	After the stroke	1. Cerebral infarction volume 2. mNSS 3. Foot fault	1. ↓, *p* < 0.05 2. ↓, *p* < 0.05 3. ↓, *p* < 0.05
Kim et al., 2007	Male Sprague Dawley rats (240–260 g)	MCAO	C: NS, NR, NR E: SB, 300 mg/kg, i.p.	After the surgery for 24 h	1. P-AKt	1. ↑, *p* < 0.05
Tu et al., 2017	Male Sprague Dawley rats weighing 250 ± 20 g	MCAO	C: NS, NR, NR E: SB, NR, i.p.	After the stroke for 7 d	1. Morris water maze	1. ↓, *p* < 0.05

SD, Sprague Dawley; MCAO, middle cerebral artery occlusion; C, control group; E, experimental group; NR, no report; NS, normal saline; SB, sodium butyrate; i.p., intraperitoneal injection; h, hour; d, day; min, minute; ↑, the indicator rose; ↓, the indicator declined; P, statistical significance.

### 3.3 Analysis of quality evaluation methods

**Figure 2:**
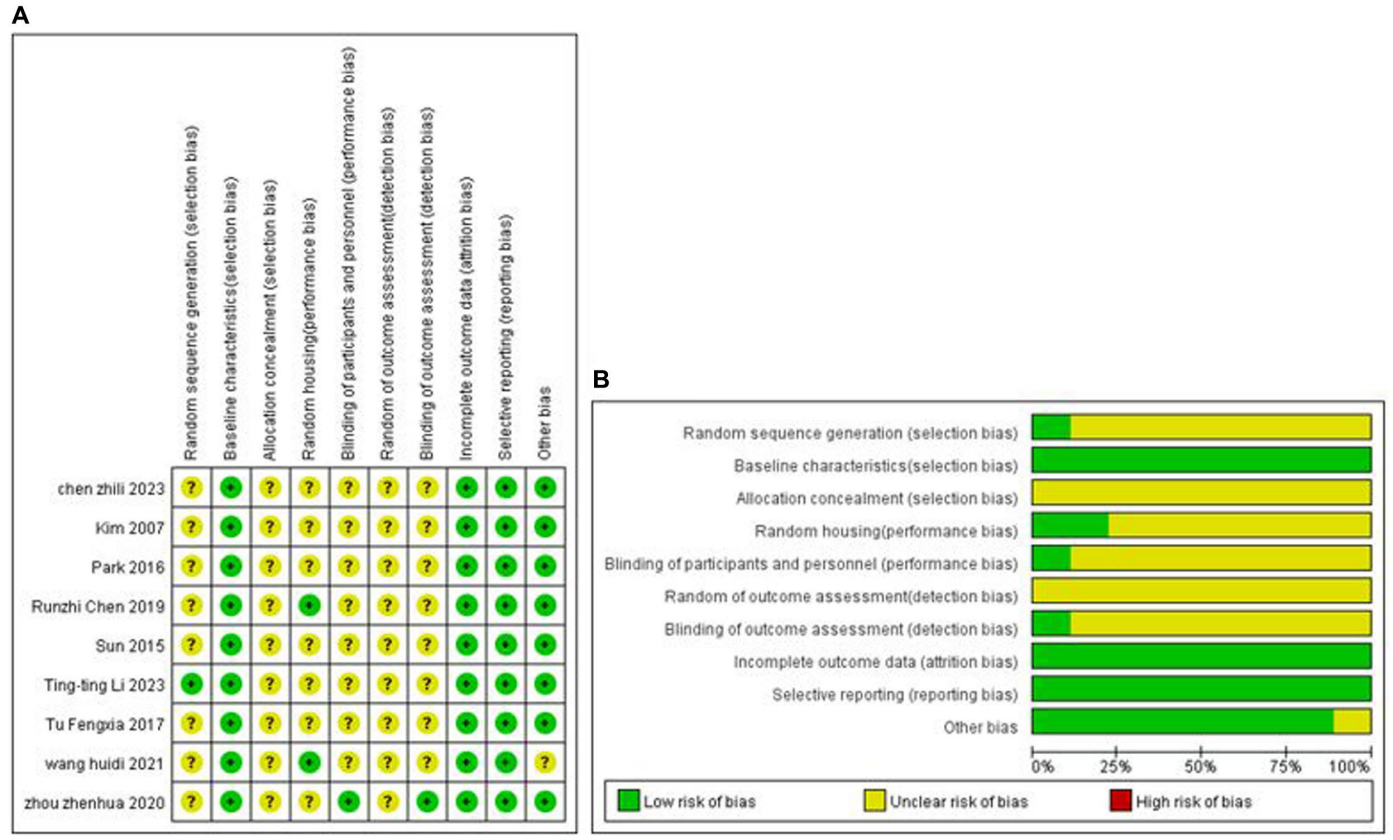
Methodological quality assessment of the risk of bias. **(A)** Risk of bias summary: review authors’ judgment of each risk of bias item for each included study. **(B)** Risk of bias plot: Review authors’ judgment for each risk of bias item, expressed as a percentage across all included studies.

One study explicitly used the random number table grouping method, and the remaining studies mentioned only the term randomised grouping. All analyses were adjusted for baseline characteristics. None of the studies mentioned allocation concealment; two studies mentioned placement randomisation, and 1 study indicated blinding. The study-specific risk of bias plots for the included studies are shown in Figure 2. Some of the data from seven studies were extracted from the images by GetData Graph Digitizer software and may be biased.

### 3.4 Volume analysis of cerebral infarction

**Figure 3:**
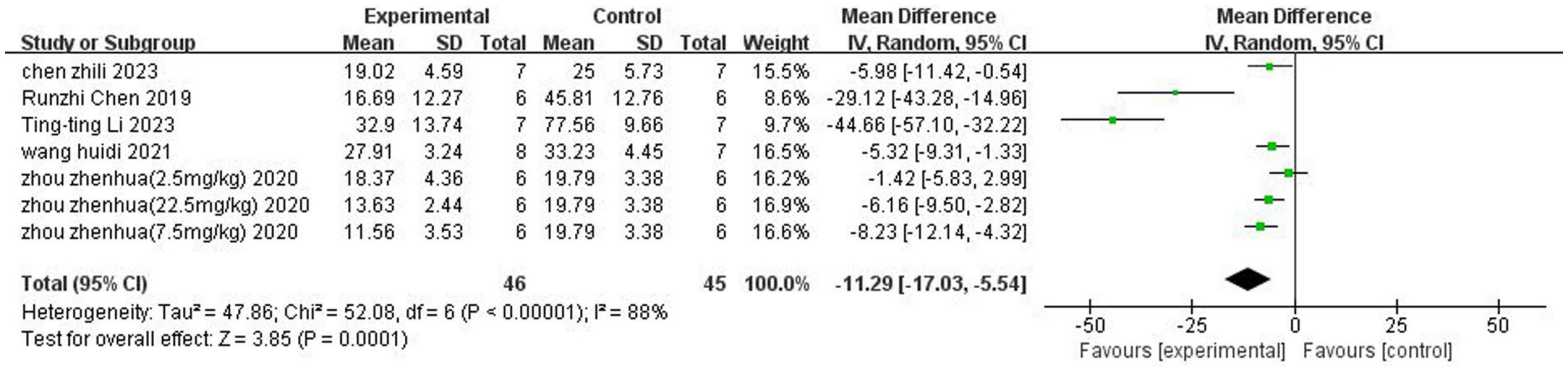
Forest plot: Reduction of cerebral infarct volume by butyrate compared with the control group.

A total of 5 (Chen et al., 2019; Wang et al., 2021; Zhou et al., 2021;Chen et al., 2023; Li et al., 2023) studies reported the percentage of cerebral infarction volume. Heterogeneity was considerable across studies (*p* < 0.00001, *I*^2^ = 88%), and a random-effects model was used. The results showed that butyrate reduced cerebral infarct volume (MD = −11.29, 95% CI = [−17.03, −5.54], *p* < 0.05). The forest plot is shown in Figure 3.

### 3.5 Neurological function score analysis

#### 3.5.1 The mNSS neurological function rating scale

**Figure 4:**
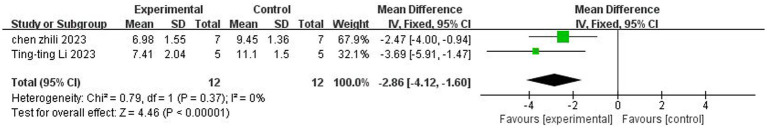
Forest plot: Effect of butyrate on reduction of mNSS score compared with the control group.

A total of two studies (Chen et al., 2023; Li et al., 2023) reported mNSS neurologic function ratings. There was no heterogeneity across studies (*p* = 0.37, *I*^2^ = 0%), and a fixed-effects model was used. The results showed that butyrate reduced mNSS scores after IS (MD = −2.86, 95%CI = [−4.12, −1.60], *p* < 0.005). The forest plot is shown in Figure 4.

#### 3.5.2 Foot fault test

**Figure 5:**
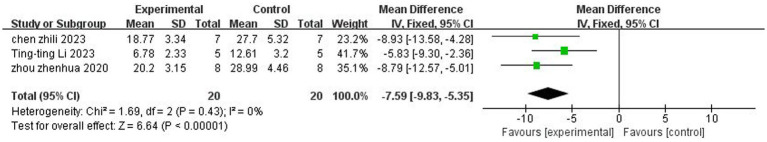
Forest plot: Effect of butyrate on reduction of foot fault score as compared to the control group.

A total of three (Zhou et al., 2021; Chen et al., 2023; Li et al., 2023) studies reported the foot fault test. There was no heterogeneity across studies (*p* = 0.43, *I*^2^ = 0%), and a fixed-effects model was used. The results showed that butyrate reduced foot fault scores after IS (MD = −7.59, 95%CI = [−9.83, −5, 35], *p* < 0.005). The forest plot is shown in Figure 5.

### 3.6 Morris water maze analysis

**Figure 6:**
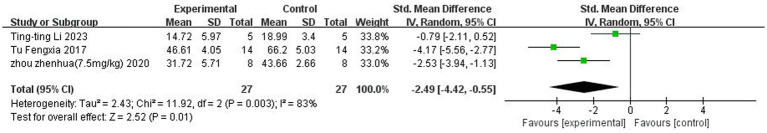
Forest plot: Effect of butyrate on reducing water maze time compared to the control group.

A total of three (Tu et al., 2017; Zhou et al., 2021; Li et al., 2023) studies reported Morris water maze. Heterogeneity was considerable across studies (*p* < 0.05, *I*^2^ = 83%), and a random-effects model was used. The results showed that butyrate reduced the time to the Morris water maze after IS (SMD = −2.49, 95%CI = [−4.42, −0.55], *p* < 0.05). The forest plot is shown in Figure 6.

### 3.7 Analysis of inflammatory factors in the brain

#### 3.7.1 IL-1β

**Figure 7:**
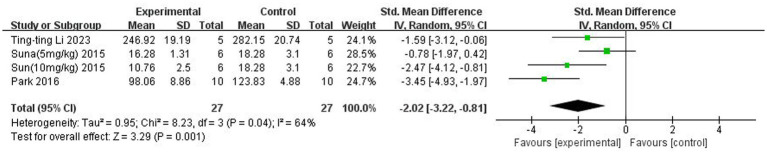
Forest plot: Effect of butyrate on lowering IL-1β in the brain compared to the control group.

A total of three (Sun et al., 2015; Park and Sohrabji, 2016; Li et al., 2023) studies reported IL-1β levels in the brain. Heterogeneity across studies was large (*p* = 0.04, *I*^2^ = 64%), and a random-effects model was used. The results showed that butyrate reduced IL-β content in the brain (SMD = −2.02, 95% CI = [−3.22, −0.81], *p* < 0.005). The forest plot is shown in Figure 7.

#### 3.7.2 TNF-α

**Figure 8:**
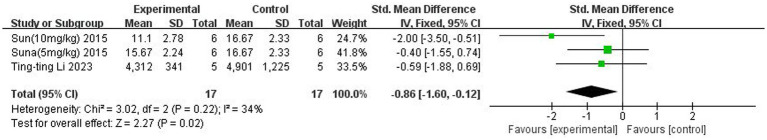
Forest plot: Effect of butyrate on reducing TNF-α in the brain compared to the control group.

A total of two (Sun et al., 2015; Li et al., 2023) studies reported TNF-α levels in the brain. Heterogeneity across studies was small (*p* = 0.22, *I*^2^ = 34%), and a fixed-effects model was used. The results showed that butyrate reduced TNF-α content in the brain (SMD = −0.86, 95% CI = [−1.60, −0.12], *p* < 0.05). The forest plot is shown in Figure 8.

### 3.8 Analysis of inflammatory factor IL-1β in peripheral blood

**Figure 9:**
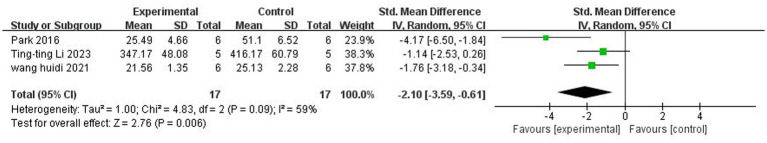
Forest plot: Effect of butyrate on lowering IL-1β in peripheral blood compared to the control group.

A total of three (Park and Sohrabji, 2016; Wang et al., 2021; Li et al., 2023) studies reported IL-1β levels in the peripheral vasculature. There was significant heterogeneity among the studies (*p* = 0.09, *I*^2^ = 59%), and a random-effects model was used. The results showed that butyrate reduced IL-1β levels in the peripheral vasculature (SMD = −2.10, 95% CI = [−3.59, −0.61], *p* < 0.05). The forest plot is shown in Figure 9.

### 3.9 BDNF analysis

**Figure 10:**
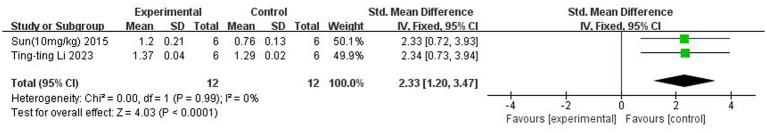
Forest plot: Effect of butyrate on elevating BDNF levels compared to the control group.

A total of two (Sun et al., 2015; Li et al., 2023) studies reported BDNF content. There was no heterogeneity across studies (*p* = 0.99, *I*^2^ = 0%), and a fixed-effects model was used. The results showed that butyrate increased BDNF content in the brain after IS (SMD = 2.33, 95% CI = [1.20, 3.47], *p* < 0.005). The forest plot is shown in Figure 10.

### 3.10 P-Akt analysis

**Figure 11:**
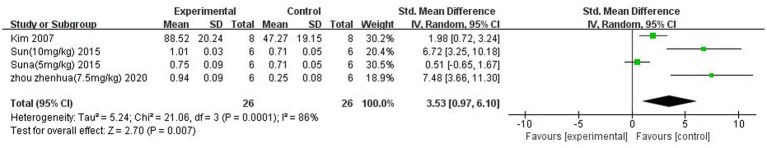
Forest plot: Effect of butyrate on P-Akt expression compared to the control group.

A total of three (Kim et al., 2007; Sun et al., 2015; Zhou et al., 2021) studies reported P-Akt expression. There was heterogeneity across studies (*p* = 0.001, *I*^2^ = 86%), and a random-effects model was used. The results showed that butyrate increased P-Akt expression after IS (SMD = 3.53 95%CI = [0.97, 6.10], *p* < 0.05). The forest plot is shown in Figure 11.

### 3.11 Sensitivity analysis

#### 3.11.1 Cerebral infarction volume

Due to the significant heterogeneity of this study, sensitivity analysis was used. After excluding (Li et al., 2023) and (Chen et al., 2019), *I*^2^ decreased from 92 to 25%. After careful analysis of each study, it was found that the butyrate treatment cycles of 14 and 28 days were much more significant in the two studies than in the other studies, which shows that different butyrate treatment cycles may produce heterogeneity. However, the results also support that butyrate effectively reduces cerebral infarction volume after IS.

#### 3.11.2 Morris water maze

Sensitivity analysis was used because of the significant heterogeneity of this study. Excluding any of the studies that did not affect heterogeneity, the results were more stable. The results support that butyrate reduces Morris water maze time after IS.

#### 3.11.3 IL-1β in the brain

Due to the significant heterogeneity of this study, sensitivity analysis was used. After excluding the risky Park (Park and Sohrabji, 2016), *I*^2^ decreased from 64 to 26%. After careful analysis of each study, the animals in this study were female rats, while all other studies were male rats. It can be seen that choosing different sexes of animals may produce heterogeneity. However, the results also support that butyrate effectively reduces IL-1β in the brain after IS.

#### 3.11.4 IL-1β in peripheral blood

Due to the significant heterogeneity of this study, sensitivity analysis was used. *I*^2^ decreased from 59 to 0% after excluding the riskier Park (Park and Sohrabji, 2016). After careful analysis of each study, the detection time in this study was the 7th day after modelling, whereas, in the other two studies, the detection was performed within 2 days. It can be seen that different testing times may produce heterogeneity. However, the results also support that butyrate effectively reduces IL-1β in peripheral blood after IS.

#### 3.11.5 P-Akt

Sensitivity analysis was used because of the significant heterogeneity of this study. Excluding any of the studies did not affect the heterogeneity, and the results were more stable. The results support that butyrate can increase P-Akt expression after IS.

### 3.12 Subgroup analysis

**Figure 12:**
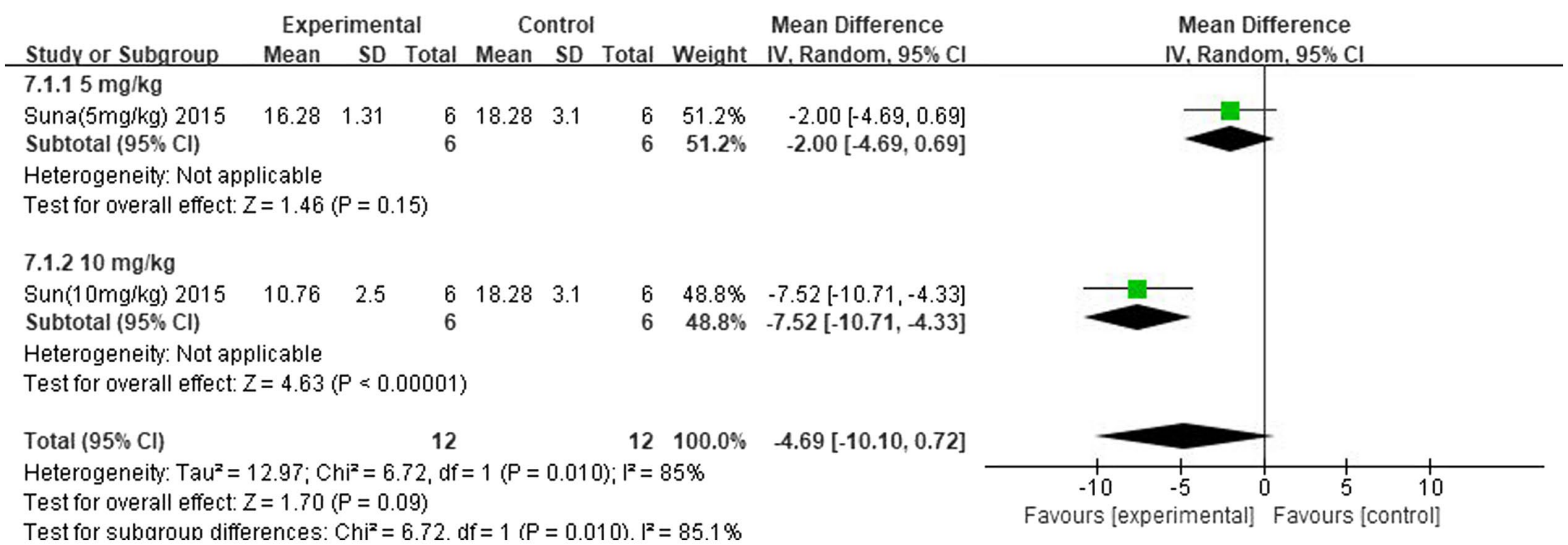
Forest plot. Subgroup analysis of the robust feeding method.

**Figure 13:**
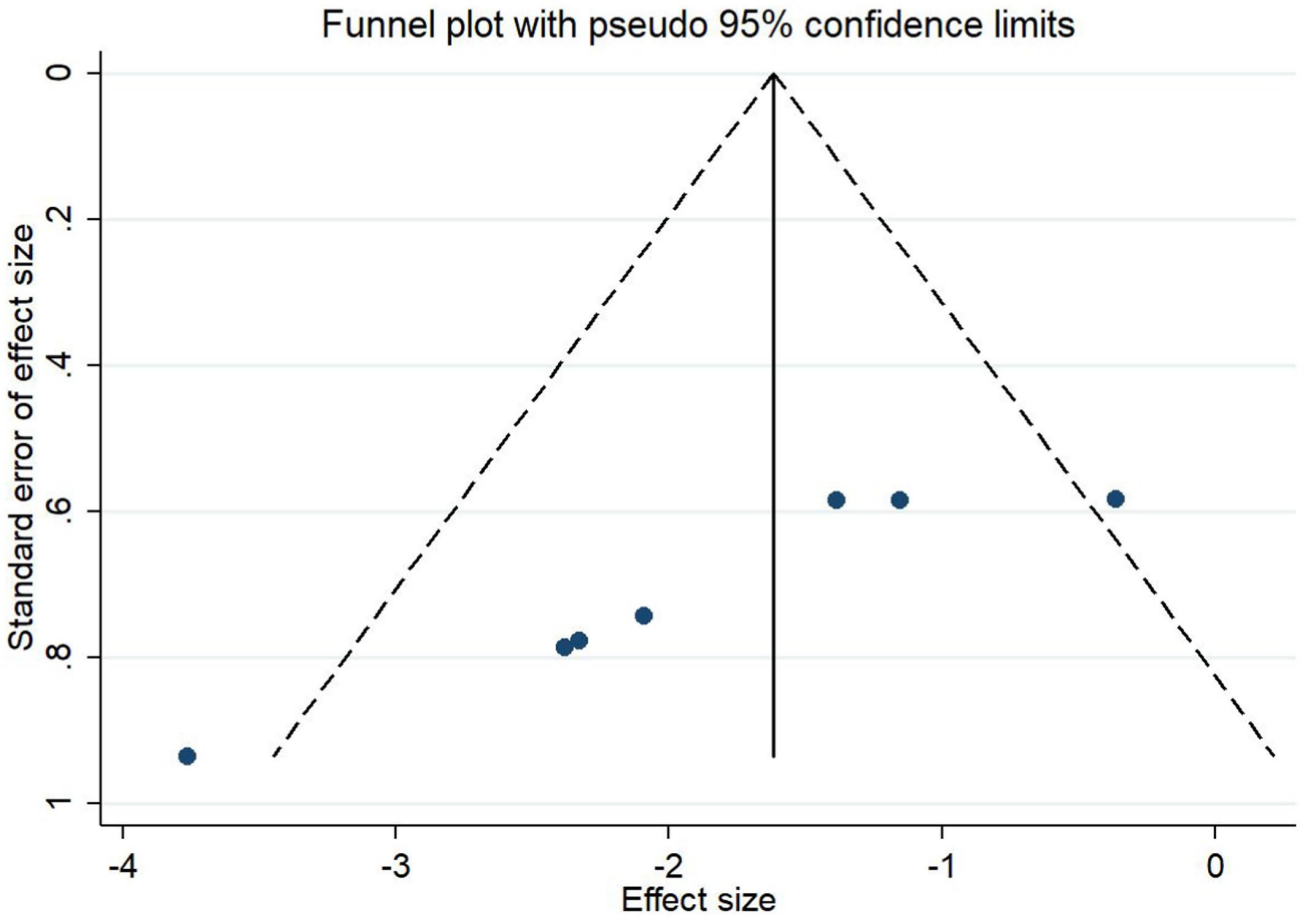
Forest plot. Nasal injection subgroup analysis.

The improvement of IS was analysed separately for different concentrations of the strong-feeding method and nasal injection according to the butyrate administration method. The subgroup analysis of the strong-feeding method is shown in Figure 12, and the concentration of 10 mg/kg was better than 5 mg/kg in reducing IL-1β in the brain after IS. The subgroup analysis of the nasal injection is shown in Figure 13, and the concentration of 7.5 mg/kg was better than 22.5 mg/kg in reducing the area of cerebral infarcts after IS, which was superior to that of 2.5 mg/kg.

### 3.13 Publication bias

**Figure 14:**
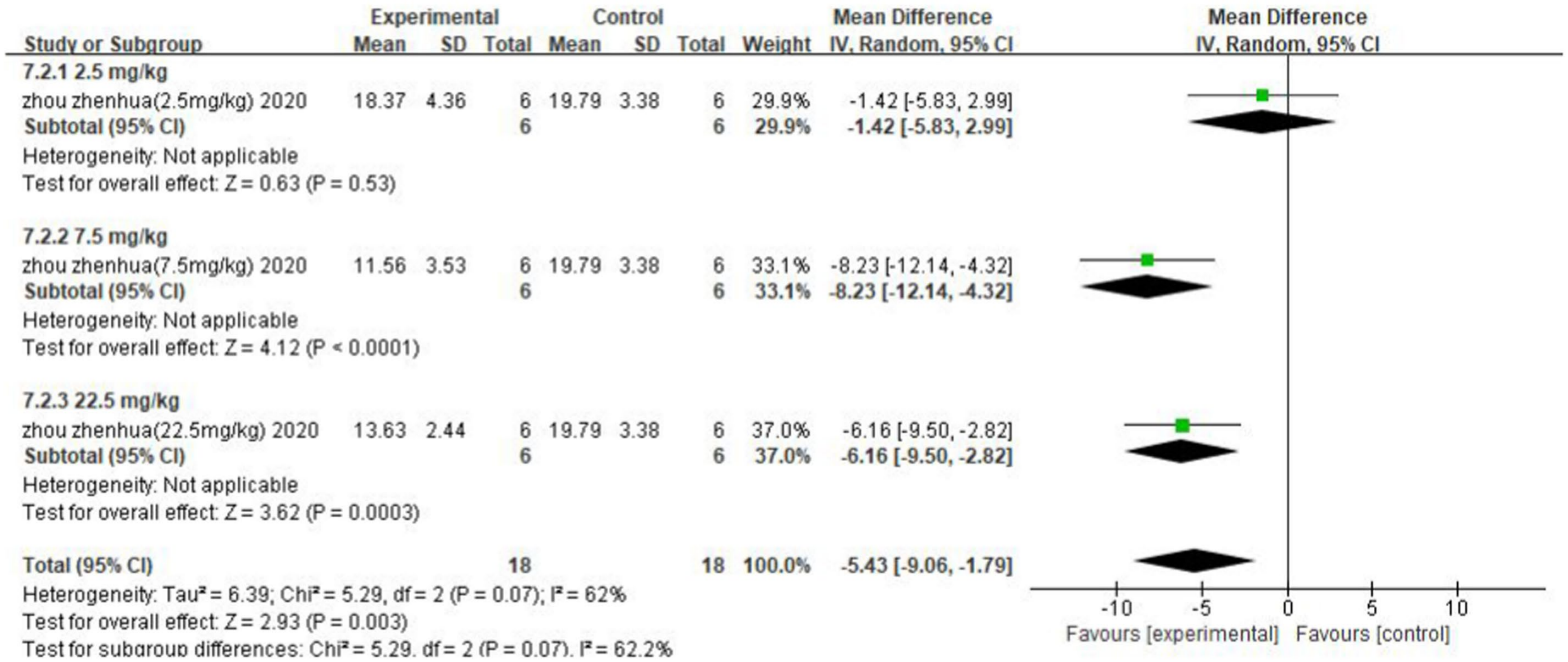
Funnel plot: Cerebral infarction volume analysis suggests publication bias.

The analysis of bias for the outcome indicator of cerebral infarction volume included five studies (seven data sets). As shown in Figure 14, the asymmetry on both sides of the funnel plot indicates the presence of publication bias. Egger’s test results indicated that *t* = 0.02 < 0.05, indicating the presence of publication bias.

## 4 Discussion

This study is the first systematic review of the neuroprotective effects of butyrate in an animal model of IS. We comprehensively evaluated the neuroprotective effects of butyrate in terms of inflammatory factors, P-Akt, BDNF, cerebral infarct volume, and neurological function scores. We found that butyrate could exert neuroprotective effects in IS animal models by suppressing intracerebral and peripheral inflammatory responses, inhibiting apoptosis, and increasing neurotrophic factors in the brain.

IS is caused by an interruption in the blood supply to an area of the brain. This produces an area of hypoxia and glucose deprivation. Dead cells in this region release various danger-associated molecular patterns, DAMPs, which stimulate the production of inflammatory cytokines and chemokines. Microglia in the brain are encouraged to rapidly activate and polarise into the M1 subtype, which elicits an inflammatory cascade response and secretes various inflammatory factors and chemokines, such as TNFα and IL-1β. It has been shown that IL-1β is rapidly upregulated in the brain in response to IS (Nguyen et al., 2016). IL-1β exerts neurotoxic effects in IS, and blocking its products has been shown to reduce IS neurological damage (Weber et al., 2010). TNF-α is expressed at low levels in the healthy CNS. However, after the occurrence of IS, it is increased with its two known receptors, TNFR1 and TNFR (Nguyen et al., 2016). Lowering their levels is an effective treatment for IS (Liguz-Lecznar et al., 2015). However, in peripheral blood, changes in inflammatory factors are currently controversial. Some studies have indicated increased levels of IL-1β in the blood of IS patients compared to controls (Sotgiu et al., 2006; Tuttolomondo et al., 2009; Wytrykowska et al., 2016), but others have reported no change in IL-1β levels in the blood of IS patients (Emsley et al., 2007; Ormstad et al., 2011). As most of these studies showing increased IL-1β were tested within 72 h, the difference in results may be due to differences in the timing of blood draws. In recent years, it has been found that IS also causes damage to the intestinal mucosa (Zhang et al., 2021). Damaged intestinal mucosa leads to the entry of the bacterial product of the gut, lipopolysaccharide (LPS), into the circulatory system (Wang et al., 2022), which activates the LPS/TLR4/NF-κB pathway leading to increased levels of IL-1β in the blood. In conclusion, both cytokines have a role in ischaemia-induced neuronal injury preceded by an early inflammatory response that exacerbates nerve damage (Basu et al., 2005). Thus, IL-1β and TNF-α play critical roles in the pathogenesis of IS and are potential targets for IS therapy.

In the present study, we showed by analysis that butyrate can effectively attenuate the levels of TNFα and IL-1β in the brain and the level of IL-1β in the blood of IS animals. Within the brain, butyrate inhibited microglia activation and reduced the secretion of inflammatory factors in rats (Park and Sohrabji, 2016). Li et al. (2023) found that butyrate regulated microglia phenotype through Treg cells, thereby reducing TNFαIL-1β levels. In addition, in SCFA, butyrate was the most effective in inhibiting the activity of HDAC (Turner and Lupton, 2011; Flint et al., 2012). Butyrate inhibits HDAC recruitment to the promoter via transcription factor-specific protein 1/specific protein 3 (Sp1/Sp3), leading to histone hyperacetylation. Many of the anticancer activities of butyrate are mediated by the inhibition of HDAC, including cell proliferation, cell differentiation or apoptosis, and induction or inhibition of gene expression (Donohoe et al., 2012). In addition to its role as an antitumor agent, butyrate partially achieves its anti-inflammatory effects by inhibiting HDAC (Park et al., 2015). For example, butyrate reduced neuroinflammation caused by microglia in IS mice by inhibiting HDAC, thereby altering the gene initiation of histone-3-lysine 9-acetylation (H3K9ac) in microglia (Patnala et al., 2017). In conclusion, butyrate modulates microglia in the brain to attenuate neuroinflammation in IS through multiple mechanisms. However, the anti-inflammatory effects of butyrate in the blood are more complex. On the one hand, peripheral immune changes in the blood are mainly alterations in acquired immunity, manifested as changes in T-cell subsets. Butyrate can regulate peripheral immunity through Tregs/TGF-β/T cells, which significantly increases the ratio of CD4+/CD8+ T cells, thus improving peripheral inflammation (Li et al., 2023). On the other hand, butyrate could repair and enhance the function of the intestinal epithelial barrier, thereby reducing the inflammatory response induced by LPS entering the circulation of the body (Huang et al., 2015). MUC2 protein produced by cuprocytes in the intestine is the most prominent protein in the intestinal mucosal layer and can strengthen it (Canani et al., 2011). Zhang et al. (2021) found that the intestinal MUC2 mRNA transcript level was significantly reduced after brain injury. Butyrate can increase the expression of MUC2 protein and enhance the protection of the intestinal barrier of Caco-2 monolayer cells (Elamin et al., 2013). Butyrate also increases the expression of trefoil factor (TFF) (a mucin-related peptide), which maintains and repairs the intestinal mucosa (Hamer et al., 2008; Leonel and Alvarez-Leite, 2012). In addition, butyrate regulates the expression of compact proteins and reduces paracellular permeability (Matter et al., 2005; Meijer et al., 2010).

**Figure 15:**
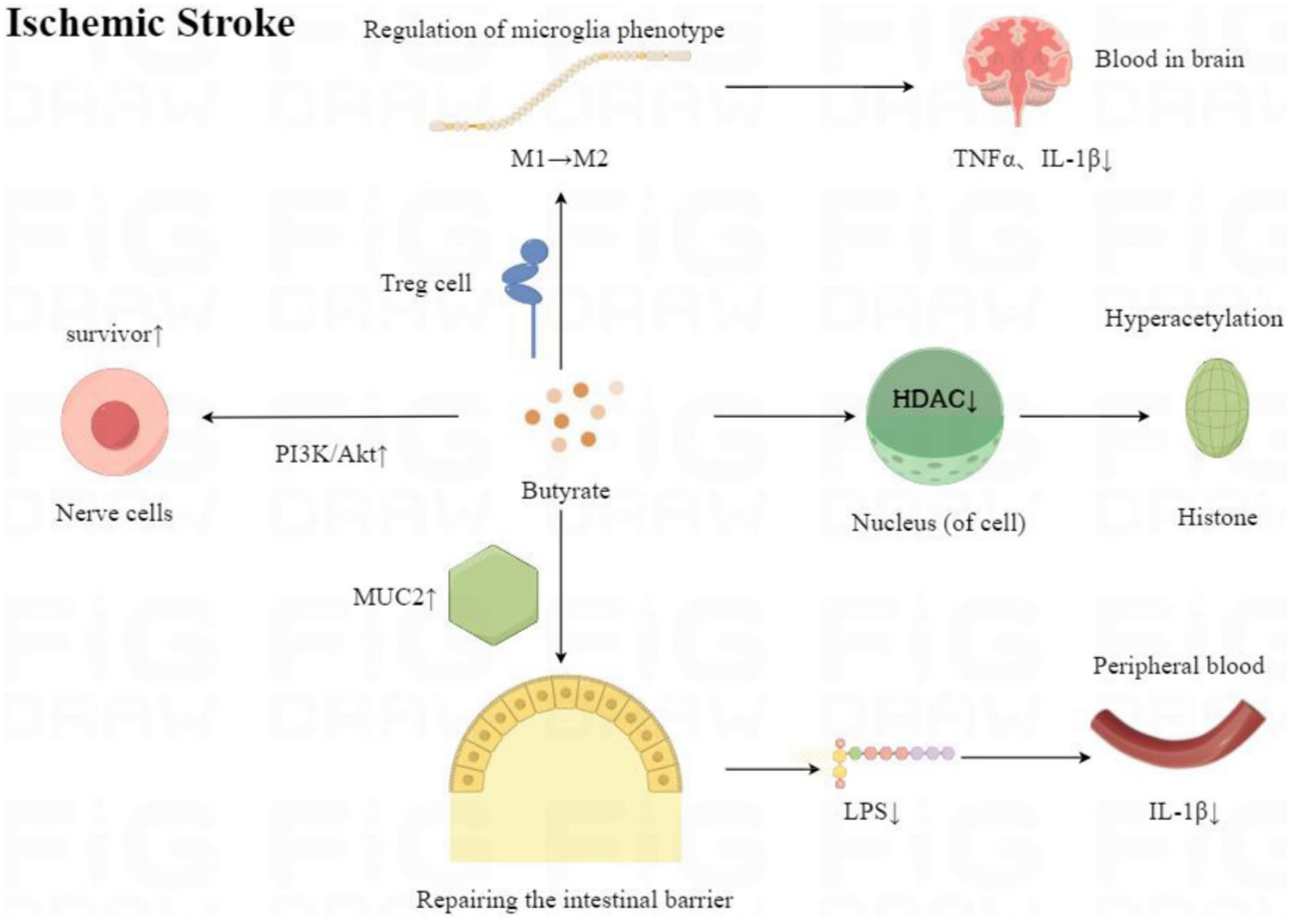
Mechanisms of neuroprotective effects of butyrate after ischaemic stroke onset.

In the acute phase of IS, neuronal apoptosis is reversible in the ischaemic hemi-dark band. Therefore, salvaging the ischaemic semi-dark bar is the most effective treatment for IS. The phosphatidylinositol 3-kinase/protein kinase B (PI3K/Akt) pathway is critical for neuronal cell survival after IS (Kilic et al., 2017). In this study, we demonstrated by analysis that butyrate can effectively increase P-Akt expression, thereby reducing nerve damage. In addition, the present study showed that butyrate reduced cerebral infarct volume and mNSS, Foot fault score, decreased Morris water maze time, and increased BDNF content. Therefore, the present study reveals to some extent that butyrate can protect the animal brain from IS damage, as in Figure 15, and improve the neurological function of animals.

It has been shown that butyrate can also ameliorate neurological injury in IS through other mechanisms. IL-22 can positively regulate angiogenesis by binding to the IL-22R receptor on the surface of endothelial cells (Hu et al., 2018). Butyrate can reduce neuronal apoptosis by inducing vascular remodelling through IL-22 in IS-aged mice to attenuate nerve injury (Chen et al., 2023). In addition, after the onset of IS, apoptosis, neuroinflammation, and altered expression of TJ-related proteins cause damage to the blood–brain barrier (BBB) (Khatri et al., 2012), exacerbating the impairment of neurological function. A recent study found that the endothelial glycocalyx layer (the first line of defence to protect the BBB) was relatively thicker in the brains of butyrate-treated IS mice than distilled water (Wang et al., 2021). Wang et al. (2021) also found that the level of matrix metalloproteinase-9 (MMP-9) was significantly reduced in the brains of butyrate-treated stroke mice. In brain tissue, MMP-9 plays a crucial role in the BBB. In response to inflammatory factors, MMP-9 can be activated and secreted, leading to proteolytic damage of extracellular matrix components and degradation of basement membrane and TJ proteins (Zhu et al., 2018; Qin et al., 2019). Both *in vivo* and *in vitro* data suggest that low expression or downregulation of MMP-9 activity restores the BBB and increases TJ protein expression (Yang et al., 2007; Zhu et al., 2018; Qin et al., 2019). Thus, butyrate could alleviate the nerve damage in IS by repairing and improving the BBB. However, the pathophysiologic mechanisms of IS are complex, with overlapping and interacting links. The protective effect of butyrate on IS needs further study.

Subgroup analyses in the present study revealed differences in the neuroimproving effects of different butyrate concentrations on IS. Previous *in vitro* studies have shown that low concentrations (≤2 mm) of butyrate promote intestinal barrier function, but high concentrations (5 or 8 mm) of butyrate may be disruptive (Peng et al., 2009). Currently, the commonly used dose for rectal administration in clinical practice is 100 mm butyrate which is comparable to the physiological concentration in the human colon after consumption of a high-fibre diet (Wang et al., 2012). Wang et al. (2021) found that 100 mm of butyrate increased the concentration of butyrate in the faeces of IS mice and was influential in improving neurological function after IS. However, the most effective butyrate concentration for treating IS in clinical trials is still worth exploring.

Limitations of this systematic evaluation are listed as follows: (1) The included studies measured a wide range of neuroimmune responses. However, the few studies measuring these outcomes precluded a meta-analysis of most neuroimmune responses. (2) There was significant heterogeneity among the included studies. Different modelling methods, as well as animal breeds, were used in the studies, and in addition. However, butyrate was used in all intervention groups; there were differences in butyrate concentration, dose, and mode of administration. (3) Only one of the nine studies involved female animals, and the small number of studies did not allow for reliable subgroup analyses of sex. (4) The bias risk assessment showed that most of the bias risk criteria in the included studies were rated as unclear. This made it difficult to determine differences in results due to source bias. Future animal studies should follow the ARRIVE Animal Analysis Reporting Guidelines to ensure greater clarity in all methods (Kilkenny et al., 2011).

## 5 Conclusion

In conclusion, this review reveals that butyrate has a wide range of positive modulatory effects on neuroinflammation in animal models of IS and provides a relevant evidence-based basis for the future development of the clinical application of butyrate in treating IS. However, given that the effects of butyrate are affected by concentration, there is no literature on the optimal concentration range for IS, and it is suggested that future studies should fill this gap. In addition, the inclusion of literature in this review was slight and subject to publication bias, so further studies are needed.

## Data availability statement

The original contributions presented in the study are included in the article/supplementary material, further inquiries can be directed to the corresponding author.

## Author contributions

SY: Data curation, Software, Writing – original draft, Writing – review & editing. QJ: Writing – original draft, Writing – review & editing. JD: Writing – original draft, Writing – review & editing. ZL: Writing – review & editing. WW: Writing – review & editing. HL: Writing – review & editing. LL: Writing – review & editing, Data curation, Supervision. CM: Data curation, Supervision, Writing – review & editing. DL: Data curation, Supervision, Writing – review & editing. ZH: Data curation, Supervision, Writing – review & editing. SA: Conceptualization, Writing – review & editing.
